# Role of Treg cells in diabetes mellitus and association with traditional Chinese medicine: a literature review

**DOI:** 10.3389/fimmu.2026.1763798

**Published:** 2026-03-25

**Authors:** Weiran Liu, Rong Liu, Zhongming Wu, Yong Zhang

**Affiliations:** 1 The First Clinical College of Shandong University of Traditional Chinese Medicine, Jinan, China; 2 Endocrine and Metabolic Diseases Hospital of Shandong First Medical University & Shandong Academy of Medical Sciences, Jinan, China; 3 Shandong First Medical University & Shandong Institute of Endocrine and Metabolic Diseases, Jinan, China

**Keywords:** arteriosclerotic cardiovascular disease, diabetic nephropathy, diabetic peripheral neuropathy, diabetic retinopathy, regulatory Treg cells, traditional Chinese medicine, type 1 diabetes, type 2 diabetes

## Abstract

Regulatory T cells (Tregs) are essential for immune tolerance, and their dysfunction is implicated in diabetes mellitus (DM) and its complications. This review examines the role of Tregs in type 1 and type 2 diabetes (T1DM, T2DM) and the potential of traditional Chinese medicine (TCM) in modulating Treg responses. Tregs contribute to β-cell autoimmunity in T1DM, inflammation and insulin resistance in T2DM, and complications such as nephropathy and vasculopathy. Recent studies show that TCM-derived compounds, including polysaccharides, flavonoids, and alkaloids, enhance Treg function, restore Treg/Th17 balance, and improve immune homeostasis. Tregs are a key link between immune regulation and metabolic dysfunction. TCM offers promising strategies for Treg-targeted immunomodulation, supporting integrative approaches to the prevention and treatment of DM and related immune disorders.

## 1 Introduction

Diabetes mellitus (DM) is a common, potentially devastating disease of the endocrine system, and its prevalence has been increasing over the past few decades. By 2030, DM is predicted be the seventh leading cause of death worldwide (1). With the gradual overall increase in wealth worldwide and quality of life, intake of sugar and fat is also increasing, and the number of people with DM is expected to reach 783.2 million by 2045 (2).

The pathogenesis of DM is complex; involving various genetic, viral, metabolic, and autoimmune factors, as well and geographic and environmental factors (3, 4). Diabetes is classified into two types: type 1 (T1DM) and type 2 (T2DM). Studies also indicate that the prevalence of gestational diabetes has increased dramatically over the past year, affecting 9%–25% of pregnancies (5). T1DM is caused by autoimmune dysfunction of pancreatic β islet cells, resulting in an absolute deficiency of insulin (6). T2DM is mainly caused by insufficient insulin secretion due to decreased function of pancreatic β islet cells, which can no longer cope with the increased insulin demand (7). In addition to well-known complications of DM, such as diabetic nephropathy, diabetic retinopathy, peripheral neuropathy, and vascular disease, other complications such as cognitive impairment, affective disorders, and sleep disorders have also been identified, bringing new burdens and challenges to the treatment of DM (8). Recent studies have found that Tregs exert certain effects on DM and its complications. In patients with DM, Tregs can alleviate inflammation and insulin resistance by promoting a pro-inflammatory environment and secreting the anti-inflammatory cytokine interleukin-10 (IL-10), thereby alleviating inflammation and insulin resistance and metabolic disorders (9–11). In addition, Tregs have been shown to be one of the important entry points for DM by modulating insulin sensitivity (12).

The rational prescription system of traditional Chinese medicine (TCM) for the treatment of immune-related diseases can be traced back to the *Treatise on Febrile and Miscellaneous Diseases*. Many of the diseases described in the book have symptoms similar to those of immune diseases, such as water Qi disease, which are similar to the symptoms of nephritis or nephrotic syndrome in modern medicine (13). Modern research has found that TCM that regulate immune mechanisms can provide new insights into various diseases to improve their clinical treatment effects. In recent years, major breakthroughs in TCM have elucidated their specific mechanisms of action, with Tregs as a breakthrough point. Given the importance of this research, it is of great scientific and theoretical significance to study the relationship between the active ingredients of TCM and Tregs, as well as the regulatory effects of TCM on the immune system. This review examined the role of Tregs in DM and the effects of TCM on Tregs (14, 15). Regulatory T lymphocytes (Tregs), has significant impacts and regulatory effects on diabetes and its complications. In traditional Chinese medicine, related research on it has been continuously deepening, and its significance is gradually becoming more apparent (16).

## 2 Regulatory T lymphocytes

**Figure 1:**
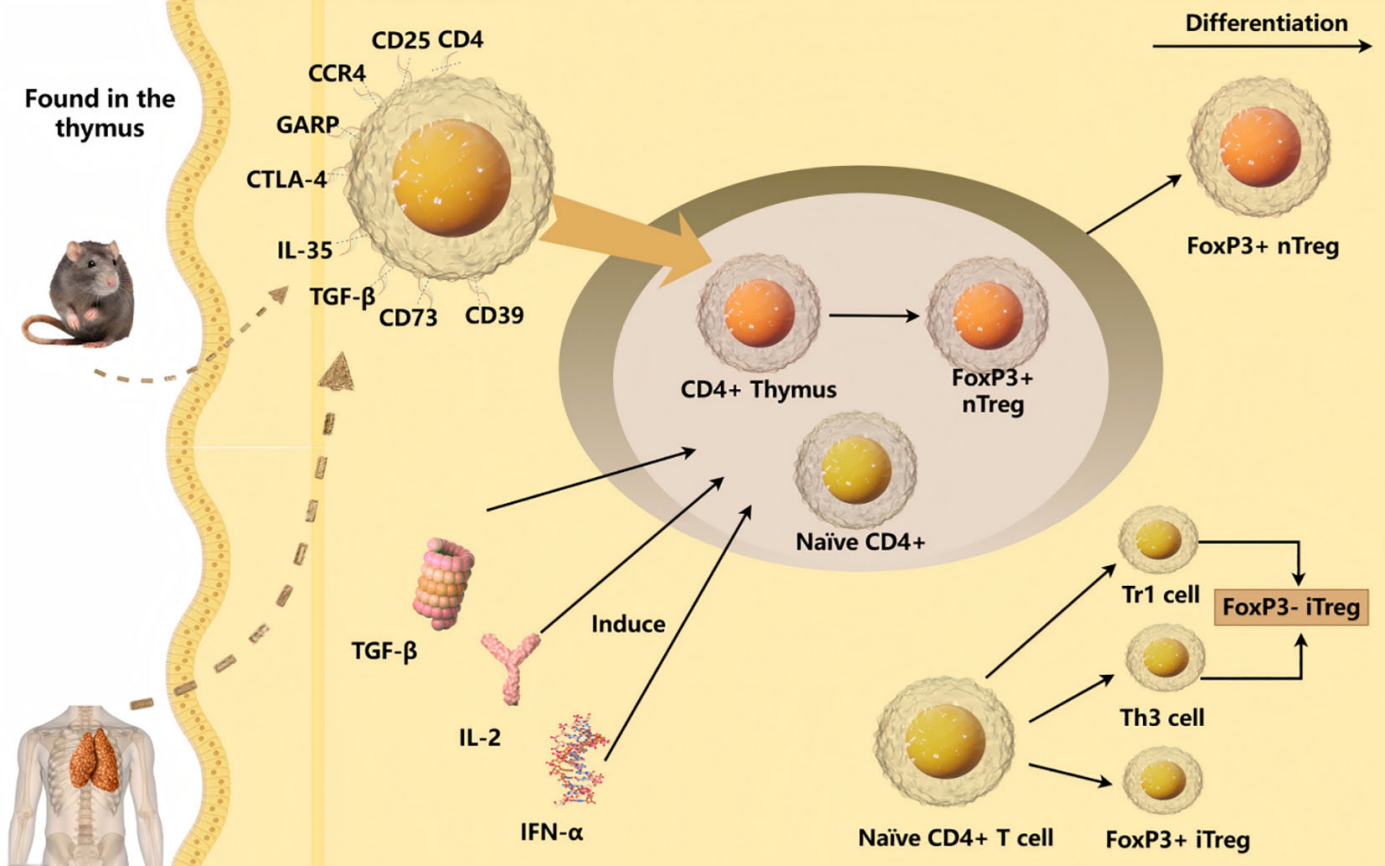
Schematic diagram of the markers, production, and differentiation of Treg cells. Treg cells have multiple markers such as CD4, CD25, CCR4, GARP, CTLA-4, IL-35, TGF-β, CD73, and CD39. Under the induction of immunosuppressive cytokines TGF-β, IFN-α, IL-2 and IL-10, and at the same time stimulated by specific antigens, peripheral mature T cells often differentiate into iTreg cells.

Tregs, which are T lymphocytes that express CD25 and suppress various infectious and autoimmune diseases, were first discovered in mice. Subsequent studies in humans showed that Tregs were mainly produced by the thymus, exported to the periphery, and widely distributed throughout the body. Tregs are broadly divided into two categories based on their origin and differentiation: natural Tregs (nTregs) and induced Tregs (iTregs) (17). Tregs are a special subset of T lymphocytes that have a low response to adaptive immunity; mainly express CD4, CD25, and Forkhead Box P3 (FoxP3); and exert anti-inflammatory effects by producing the inhibitory cytokine IL-10 to prevent immune overdose (18, 19). FoxP3 plays a key role in the development and activation of Tregs (20). FoxP3 is thought to not only function as a transcriptional repressor but also interact with other transcription factors to maintain Treg identity and function (21, 22). In addition, ectopic expression of FoxP3 can stimulate the inhibitory power of traditional T cells to a certain extent (17). Yan et al. found that in the absence of pro-inflammatory cytokines, naïve CD4+ T cells do not differentiate into Th17 cells, but instead begin to differentiate into Tregs, resulting in autoimmune impairment (23). Treg markers, production, and differentiation are summarized in **Figure 1**.

Tregs play a central role in immune homeostasis. Depletion of Tregs led to impaired tolerance and self-balance, thereby inducing autoimmune diseases (24). Tregs have numerous functional mediators, such as IL-10, TGF-β, IL-35, CD39, CD73, GARP and CTLA-4 (25). CTLA-4, a key molecule in Tregs, helps to downregulate CD80 and CD86 in dendritic cells, thereby inhibiting activation of effector cells (26). Zhou et al. found that Tregs lost FoxP3 expression during the development of DM, while gaining effector functions similar to those of effector T cells, and an increase in IFN-γ-producing Tregs was also observed in patients with DM (27, 28). TGF-β, IL-10, and IL-35, the main regulatory cytokines released by Tregs, are also involved in the corresponding inhibition (29). Tregs are also characterized by their inability to produce IL-2, which is essential for the proliferation and differentiation of effector T cells, and as a result, there are certain limitations to the inhibition mediated by IL-2 (30). Because the transcription factor T-bet is required for controlling Th1-mediated inflammation, the absence of T-bet in Tregs also causes Th1-related autoimmune diseases (31).

Thus, in regulating immunity, Tregs control the inflammatory response and adapt to the local environment by maintaining FoxP3 expression and upregulating certain transcription factors (32). Regarding Th2-mediated inflammation, Tregs inhibit the development of inflammation through self-mediated expression of the transcription factors IRF4 or GATA3 (33, 34). In contrast, the T helper cell-like Treg phenotype is reversible and typically has a demethylated TSDR at the FoxP3 site (28). Overall, CD4 is a surface marker of helper T cells, determining their differentiation into different functional subgroups (such as pro-inflammatory or immune-assisting), reflecting functional diversity; CD25 is the alpha chain of the IL-2 receptor, which can be expressed in both activated T cells and Tregs, suggesting an activated or regulatory state; FoxP3 is a key transcription factor of regulatory Tregs, determining their immunosuppressive function and being a core molecule for stabilizing immune tolerance. Thus, in regulating immunity, Tregs control the inflammatory response and adapt to the local environment by maintaining FoxP3 expression and upregulating certain transcription factors (32). Regarding Th2-mediated inflammation, Tregs inhibit the development of inflammation through self-mediated expression of the transcription factors IRF4 or GATA3 (33, 34). In contrast, the T helper cell-like Treg phenotype is reversible and typically has a demethylated TSDR at the FoxP3 site (28). Overall, CD4 is a surface marker of helper T cells, determining their differentiation into different functional subgroups (such as pro-inflammatory or immune-assisting), reflecting functional diversity; CD25 is the alpha chain of the IL-2 receptor, which can be expressed in both activated T cells and Tregs, suggesting an activated or regulatory state; FoxP3 is a key transcription factor of regulatory Tregs, determining their immunosuppressive function and being a core molecule for stabilizing immune tolerance. Tregs play a crucial role in limiting the development of T1DM. Their activation or application is more effective in the early stage of the disease, and have a promising clinical application prospect. However, long-term observation of a large number of patients is required. The main reason might be that the Tregs therapy can restore immune tolerance and prevent autoimmune activation (0000) (0000). Clinical investigations also revealed that the levels of CD4+CD25+FOXP3+ Tregs in the peripheral blood of children with type 1 diabetes were significantly reduced, and this was associated with poor glycemic control, suggesting that the defect in Tregs may lead to the disruption of immune tolerance and play a role in the pathogenesis (0000). Tregs also have a significant impact on patients with long-term type 1 diabetes who still have functional beta cells, the quality of the auto-reactive Tregs in some patients with T1DM shows an extreme bias towards the pro-inflammatory Th1 phenotype (0000) (0000). Further studies have shown that patients with TIDM who have lower levels of Tregs are more likely to develop cardiovascular complications, which may be related to CD4+CD8+FOX3+ Tregs (0000). Therefore, Tregs also play a crucial role and have significant clinical value in the clinical onset and disease regulation of type 1 diabetes.

## 3 Tregs and diabetes mellitus

### 3.1 Tregs and type 1 diabetes mellitus

T1DM is a T lymphocyte-mediated autoimmune disease that results in the destruction of insulin-producing β cells within the islets of Langerhans, and accounts for 5%–10% of all diabetes cases (35). Although patients with T1DM have shorter lifespans than healthy people, the life expectancy of patients with T1DM has increased dramatically compared to that of patients 30 years ago owing to the commercial availability of insulin (36). The detailed pathogenesis of T1DM is unclear, but it has been shown to involve a number of factors. Genetic factors may contribute to the development of T1DM, as studies have shown that syndromic diabetes is strongly associated with consanguinity. Some genetically studies have found that the incidence of T1DM is about twice as common in males than in females. However, the reason for this sex difference is unknown (37, 38). Epidemiologic, clinical, cellular, and molecular studies and animal experiments have shown that viral infections and metabolic factors are associated with the development of T1DM (39, 40). In recent decades, immunity has also become a research hotspot.

T1DM cannot be cured, and as patients age and the disease progresses, various complications can develop, including diabetic nephropathy, diabetic retinopathy, cardiovascular disease, and peripheral neuropathy (41). The main treatment is insulin, which is delivered by injection; however, some patients are prone to insulin resistance. As T1DM is characterized by the loss of self-tolerance to insulin-producing β cells in the pancreas, and Tregs are a key factor in maintaining immune self-tolerance. Studies have shown that Tregs can have a significant effect on T1DM. Tregs can delay the progression of T1DM by modulating immunity, lowering blood glucose levels, reducing islet damage and improving islet β cell function (42). Therefore, by providing a strong agonistic T-cell receptor ligand under sub-immunogenic conditions, it may be possible to promote the conversion of naïve CD4+ T cells into FoxP3+ Tregs, thereby restoring self-tolerance and effectively achieving autoimmune-specific prophylaxis. A preventive role for Tregs in T1DM has also been reported (43).

Their activation or application is more effective in the early stage of the disease, and have a promising clinical application prospect. However, long-term observation of a large number of patients is required. The main reason might be that the Tregs therapy can restore immune tolerance and prevent autoimmune activation (44). Clinical investigations also revealed that the levels of CD4+CD25+FOXP3+ Tregs in the peripheral blood of children with type 1 diabetes were significantly reduced, and this was associated with poor glycemic control, suggesting that the defect in Tregs may lead to the disruption of immune tolerance and play a role in the pathogenesis (45). Tregs also have a significant impact on patients with long-term type 1 diabetes who still have functional beta cells, the quality of the auto-reactive Tregs in some patients with T1DM shows an extreme bias towards the pro-inflammatory Th1 phenotype (46, 47). Further studies have shown that patients with TIDM who have lower levels of Tregs are more likely to develop cardiovascular complications, which may be related to CD4+CD8+FOX3+ Tregs (48). Therefore, Tregs also play a crucial role and have significant clinical value in the clinical onset and disease regulation of type 1 diabetes.

### 3.2 Tregs and type 2 diabetes mellitus

T2DM is a metabolic disease caused by ineffective function and insufficient secretion of insulin released in response to high blood sugar levels. T2DM is associated with various underlying factors, such as age, weight, genetics, and lifestyle (49). The pathogenesis of T2DM is not fully understood, but it is generally recognized that dyslipidemia, hyperglycemia, and other metabolic disorders lead to insulin resistance (IR) and β cell dysfunction through inflammation, oxidative stress, endoplasmic reticulum stress, and ectopic lipid deposition (50). Glucagon-like peptide-1 (GLP-1) receptor agonists are widely used to treat T2DM, as these agonists improve insulin sensitivity, enhance β cell function, and protect cardiovascular function. In addition, studies have suggested that bariatric surgery is an underutilized intervention for patients with T2DM that is not adequately controlled by medications (51, 52). Weight loss through diet control early after diagnosis can also substantially reverse the disease. In some countries, measures that improve living standards, such as consumption of the Mediterranean diet, have been shown to reverse the trend in metabolic diseases such as T2DM because of their health benefits (53, 54).

**Figure 2:**
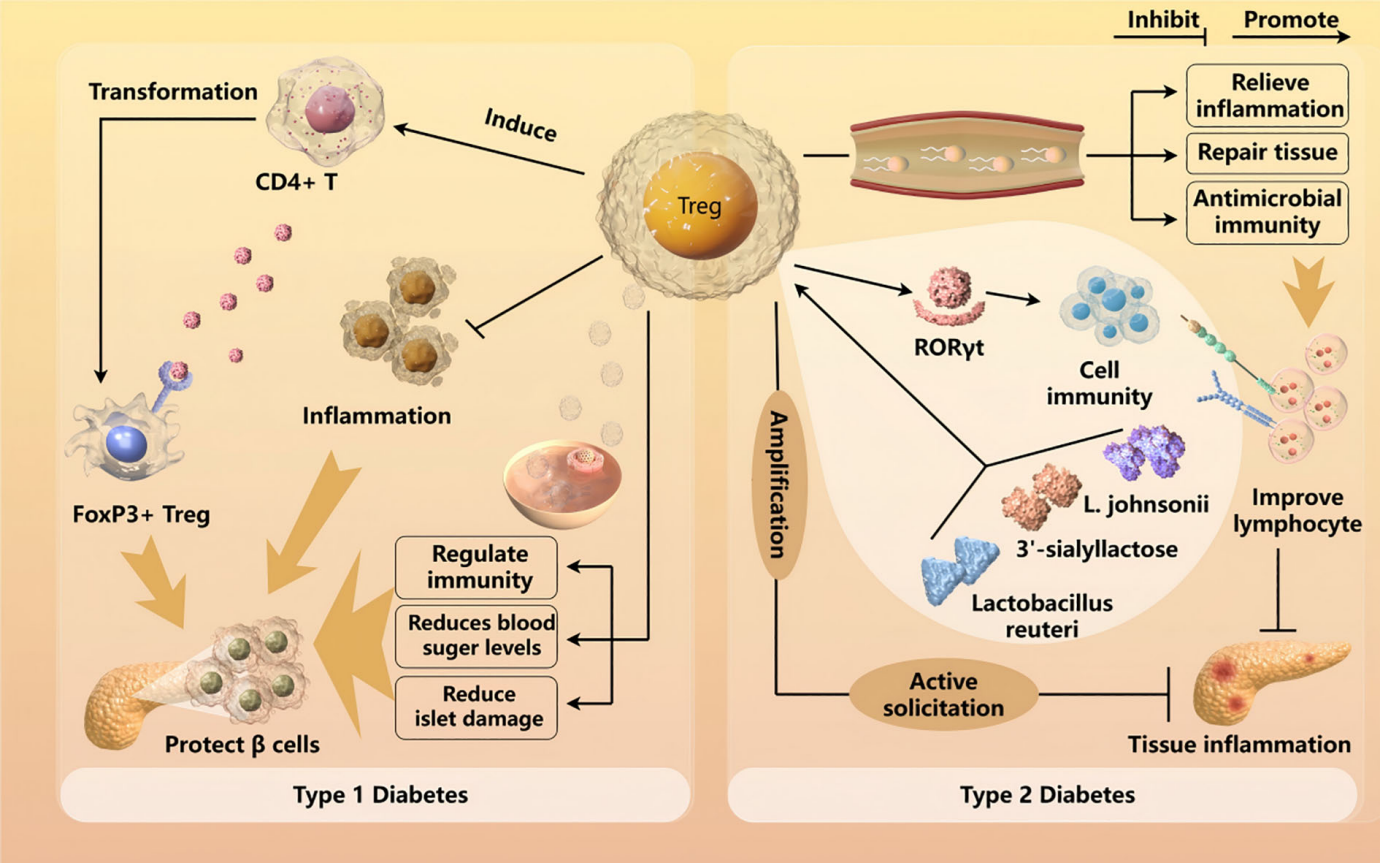
Treg cells regulate immunity to delay T1DM by lowering glucose, preserving islets, and enhancing β-cell function, crucial for immune tolerance. Inducing CD4+ T cells into FoxP3+ Tregs under subimmunogenic conditions may restore self-tolerance, preventing T1DM. In T2DM, hyperglycemia modulates Treg differentiation and peripheral markers. Tregs mitigate lymphocyte dysfunction via anti-inflammatory, antimicrobial, and reparative mechanisms, reducing visceral fat inflammation and slowing T2DM. Gut microbiota and gestational diabetes offer potential Treg-targeted therapies.

Recently, an increasing number of studies have confirmed that Tregs are associated with T2DM. High glucose levels promote the differentiation of Tregs and affect the levels of related indicators in the peripheral blood of patients with T2DM. Additionally, the anti-inflammatory, anti-microbial, and tissue repair-promoting effects of Tregs can improve the abnormal state of lymphocytes (55). Vasanthakumar et al. found that local expansion and active recruitment of Tregs can also mediate male-specific feedback, limiting the degree of inflammation in visceral adipose tissue, and thus effectively alleviating the progression of T2DM (56). Relevant *in vivo* experiments showed that treatment with 3’-sialyllactose, *Lactobacillus reuteri*, and *L. johnsonii* can promote proliferation of intestinal Tregs and regulate the expression of the transcription factor RORγt. Reducing the allergic reaction induced by ovalbumin impacts early childhood allergic diseases caused by gestational diabetes, which may be related to promotion of the development of thymus Tregs during pregnancy and improving the function of maternal Tregs in gestational diabetes, glucose homeostasis, and metabolic weight (57, 58). Taken together, these studies suggest that Tregs could be used as future research targets for T2DM. The relationship and interaction of Treg with T1DM and T2DM are shown in **Figure 2**. Furthermore, for both T1DM and T2DM, the significance of the Treg type of immune cells in the former type of immune disorder is much greater than that in the latter type of metabolic disorder.

### 3.3 Tregs and diabetic nephropathy

Diabetic nephropathy (DN) is the most common complication of diabetes and the main cause of end-stage renal disease worldwide. Renal fibrosis is the key pathological process by which DN develops into end-stage renal disease (59). DN is currently diagnosed based on biomarkers, such as protein, lipid, gene, and metabolic markers, as well as electrical signals, imaging measurements, and the presence of cells in urine (60).

Treatment options for DN include strict control of blood sugar and blood pressure with renin-angiotensin-aldosterone system (RAAS) inhibitors, lipid-lowering drugs, weight loss, and protein restriction. The standard treatment involves controlling blood glucose and blood pressure with renin-angiotensin system blockers, reducing A1c to <7% and blood pressure to <130/80 mmHg. Resolution of proteinuria is also an important therapeutic goal (61). Tian et al. found that the specific CaMKK2 inhibitor dagliaglozin alleviated mitochondrial damage and ferroptosis and significantly improved impaired lipid peroxidation and antioxidant capacity in DN mice, thereby improving kidney injury (62). An imbalance between Th17+ cells and Tregs is important in the pathogenesis of DN. Wang et al. showed that sodium-glucose cotransporter 2 regulates the SGK1/p-FoxO1/IL-23 R axis by altering t58he Na+ content in the local environment, significantly improving the Th17/Treg imbalance and effectively inhibiting the development of DN (63, 64). Hyperoside also significantly improved the inflammatory state of DN by promoting both macrophage polarization from the M1 to M2 phenotype and the differentiation of CD4+ T cells into Tregs (65). In addition, effective DN treatments could be developed based on gut microbiota-immune cell interactions, such as the induction of CD4+ Tregs by specific gut microbiota (66, 67). Wang et al. showed that dapagliflozin has a certain protective effect against the pathogenesis of DN, which may be mediated by inhibition of SGK1 and reversing the imbalance in Tregs (68). These studies show that Tregs participate in the development of DN and play a significant regulatory role.

### 3.4 Tregs and diabetic vasculopathy

Diabetic vasculopathy (DV) is a common pathological syndrome in patients with DM that commonly affects various sizes of blood vessels, including large blood vessels and microvessels, with atherosclerosis in the heart, brain, and peripheral vascular system. Diabetes is an important risk factor for vascular diseases (69, 70) and some studies have suggested that individuals with T1DM have stronger pro-inflammatory characteristics; therefore, the incidence of diabetes-related vascular complications is higher individuals with T1DM than in individuals with T2DM (71). Whyt et al. found that in a diabetic population, an individual’s susceptibility to COVID-19 greatly increased the incidence of endothelial dysfunction with microvasculopathy, which may be caused by either direct entry of the virus into endothelial cells or indirect activation of an inflammatory cascade (72). Current treatments for DV include metformin, GLP-1 agonists, and sodium-glucose cotransporter-2 inhibitors. In addition, other potential treatments, such as peroxisome proliferator-activated receptor-gamma agonists, aldose reductase inhibitors, Nox4, glucokinase agonists, and mitochondrial energy regulators, are also being actively developed (73, 74). Tregs can improve blood circulation and promote wound healing by controlling the inflammatory response, downregulating the levels of inflammatory factors, and increasing the proportion of M2 macrophages (75, 76). Deficiency and dysfunction of CD4+CD25+FoxP3+ Tregs are common immunopathological injury mechanisms in DV. Studies have shown that the levels of Tregs, as an independent protective factor, are negatively correlated with the Gensini score. Therefore, Tregs could be used as therapeutic targets in patients with coronary heart disease complicated by T2DM (77). Zhang et al. showed that Tregs improved cardiac dysfunction, myocardial hypertrophy, and fibrosis in db/db mice, which may be related to the phosphatidylinositol 3-kinase-protein kinase B and mitogen-activated protein kinase pathways, and related reductions in inflammation, apoptosis, and oxidative stress (78). In addition, a study on the association between T1DM and Tregs in cardiovascular disease showed that patients with T1DM had fewer circulating CD4+CD25+CD127- Tregs, and interestingly, more proatherogenic CD14+ CD16+ monocytes, which effectively predicted enhanced common carotid intima-media thickness and acute cardiovascular events (79). These findings suggest that during the development of DV, Tregs play a role in promoting blood circulation and decreasing atherosclerosis, thereby delaying disease progression.

### 3.5 Tregs and diabetic peripheral neuropathy

Diabetic peripheral neuropathy (DPN), a common complication of diabetes, is characterized by nerve damage caused by high blood sugar levels, leading to symptoms such as pain, tingling, and numbness, mainly in the hands and feet, which can gradually worsen over time (80). As the condition progresses, it can cause a loss of sensation in the affected area and lead to increased muscle weakness and decreased muscle coordination. In severe cases, DPN can also cause changes in foot shape, leading to joint and bone disease (81, 82). Distal symmetric polyneuropathy is the most common type of DPN in patients with DM, and approximately 50% of patients with T2DM have this condition (80–82). Hui et al. showed that DPP4 binds to IGF2-R on the surface of Tregs, and, by activating the PKA/SP1 signaling pathway, upregulates the expression of ERp29, promotes the formation of mitochondria-associated ER membrane of Tregs, and alleviates polarization of microglia in the hippocampus towards a pro-inflammatory phenotype, thus alleviating neuroinflammation and cognitive impairment in T2DM (83). Langston et al. showed that exercise can rapidly induce the expansion of muscle Treg compartments, improve mitochondrial abnormalities, and prevent metabolic disruption and overproduction of interferon (IFN)-γ, which are potential future research targets for DPN (84). Studies have found that inducing HO-1 expression inhibited the secretion of pro-inflammatory cytokines, promoted the response of Tregs, reduced the production of reactive oxygen species, alleviated oxidative stress and inflammation, and thus improved the related indicators of DPN (76). In summary, Tregs and other related immune cells have a causal relationship with DPN and play an intermediary role, providing a theoretical basis for the development of new prevention strategies and interventions (85).

### 3.6 Tregs and diabetic retinopathy

**Figure 3:**
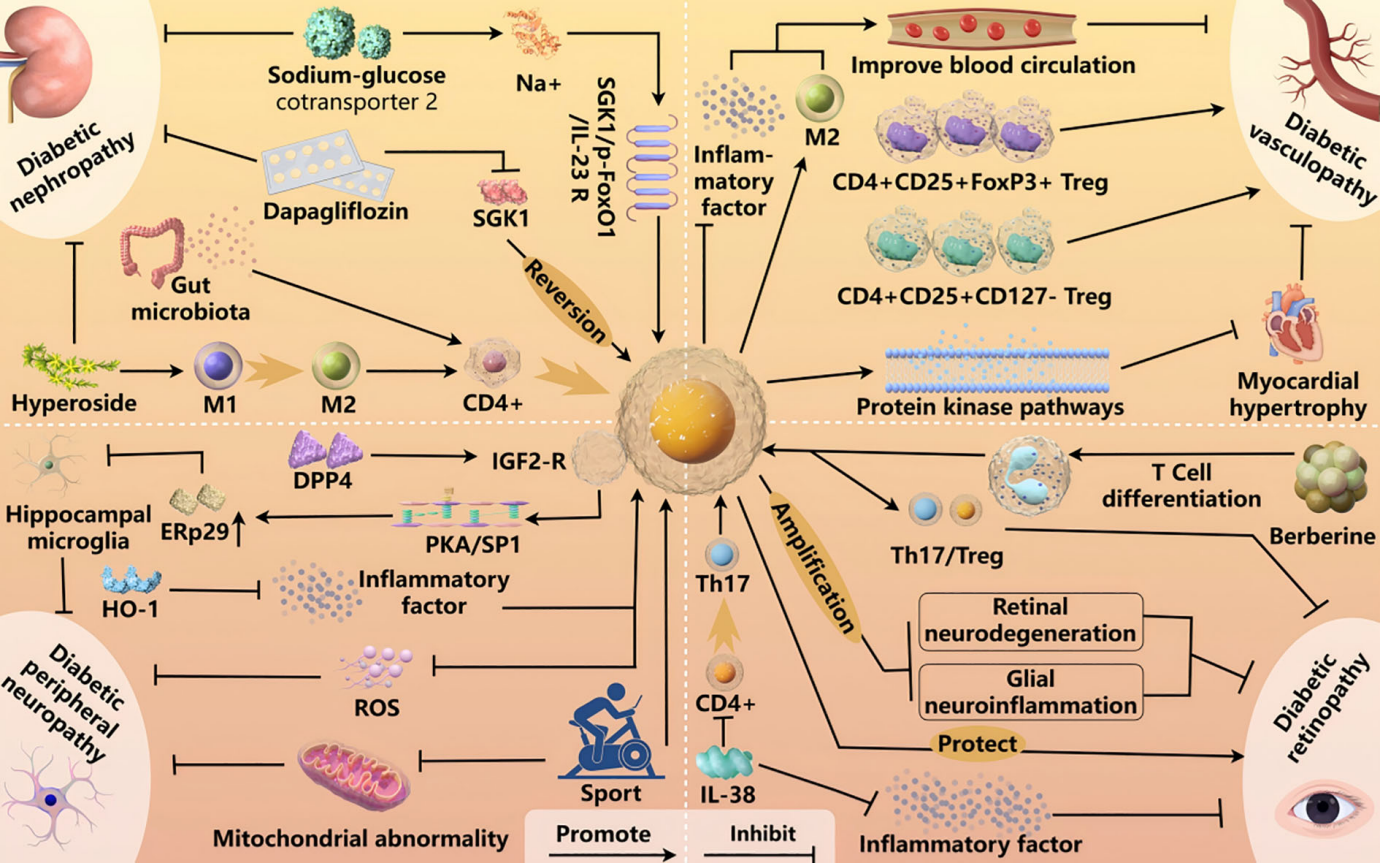
Th17/Treg imbalance drives diabetic nephropathy (DN). SGLT2 inhibitors regulate the SGK1/p-FoxO1/IL-23R axis to restore balance and inhibit DN. Hypericin modulates macrophage polarization and Treg differentiation to reduce inflammation, while gut microbiota-immune interactions offer novel DN therapies. Daglipzin protects against DN by inhibiting SGK1-mediated Treg imbalance. Tregs enhance circulation and wound healing by modulating inflammation and increasing M2 macrophages. In coronary heart disease and T2DM, Tregs mitigate myocardial dysfunction, hypertrophy, fibrosis, inflammation, and oxidative stress, predicting atherosclerosis progression. In diabetic peripheral neuropathy (DPN), DPP4 regulates Tregs via PKA/SP1 signaling to reduce neuroinflammation, while exercise and HO-1 induction improve mitochondrial function and oxidative stress, informing prevention strategies. In diabetic retinopathy (DR), berberine restores Th17/Treg balance, and IL-38 suppresses Th17 differentiation to enhance Treg-mediated immunosuppression. Treg expansion mitigates retinal neurodegeneration and inflammation, offering new therapeutic avenues for DR.

Diabetic retinopathy (DR) is an eye complication of DM caused by dysfunction of the retinal vascular structure and the vascular unit of nerve tissue, which leads to progressive damage to the corneal nerves. DR is believed to damage retinal microvessels and be one of the main causes of blindness in late stage DM. Therefore, it is often screened as part of eye exams in patients with DM (86). In recent years, evaluation, screening, imaging, and treatment of eye diseases have advanced. For example, diabetic retinopathy classification systems, such as the Early Treatment of Diabetic Retinopathy Study (ETDRS) and the International Diabetic Retinopathy Rating (ICDR) Severity Scale, can effectively predict the risk of disease progression. In addition, nanotechnologies are gaining recognition for their therapeutic potential (87, 88). Berberine can directly or indirectly regulate the differentiation of T-cell subsets, improve Treg levels, and regulate the Th17/Treg balance, which points to a potential therapeutic pathway to improve DR (89). Lai et al. showed that IL-38 further enhanced the immunosuppressive activity of Tregs by inhibiting the entry of CD4+ T lymphocytes into Th17+ T cells and significantly inhibited the inflammatory response (90). Llorian-Salvador et al. suggested that Tregs have anti-inflammatory and neuroprotective potential and could play a key role in limiting age-related retinal neurodegeneration (91). Additionally, expanding Tregs in mice alleviated changes in retinal neurodegeneration, glia, and inflammation; improved vascular permeability; and reduced neuroinflammation; suggesting a potential new treatment for preventing early DR (92). In summary, Tregs are closely associated with the prevention and treatment of DR. The interaction and mechanism of Treg with diabetic complications are shown in **Figure 3**.

## 4 Tregs and traditional Chinese medicines

According to statistics, many traditional Chinese medicines (TCM) can regulate immunity. Because of their many targets, regulatory mechanisms, and few side effects, these medicines has been widely used in recent years. The condition of the body is often improved by regulating Tregs or Treg-related immune cells, such as CD4+ and FoxP3+ cells, or by correcting the Th17/Treg imbalance (14). Different TCM belong to different meridians and have different effects and functions. When a specific immune-regulating Chinese medicine is applied to the body, it can improve its belonging meridians, so as to regulate immunity and improve the various complications of DM. At the same time, the meridians to which the drugs belong are also listed. That is: The meridians to which the drug acts, which specific organs it mainly flows through, and which specific organ it mainly exerts its effect on. For example, Chinese medicines belonging to the liver channel can be used to treat DR, and Chinese medicines belonging to the spleen channel can be used to regulate the function of the spleen and stomach. Interestingly, Chinese medicine is not simply attributed to a meridian; it can sometimes be attributed to multiple meridians to achieve a multifaceted effect (93). The following two sections provide a summary of the relationship between Tregs and some common clinical Chinese medicines and compounds used to treat DM.

### 4.1 Tregs and the active ingredients in traditional Chinese medicines

Tusizi (Dodder) belongs to the three meridians of the liver, spleen, and kidney. Hyperoside from Tusizi significantly reduced diabetic proteinuria and mesangial matrix expansion and improved fasting blood glucose levels. It also effectively regulated macrophage polarization and inhibited infiltration of renal proinflammatory macrophages (65). Danshen (Salvia) belongs to the heart and liver meridians. Salvianolic acid B, one of the main active components of Danshen, inhibited the occurrence and development of inflammation and had a protective effect against immune dysfunction caused by Treg deficiency (94). Jianghuang (Turmeric) belongs to the two meridians of the liver and spleen. Curcumin, the main component of Jianghuang, has extensive biological activities and pharmacological properties. Curcumin significantly improved the symptoms of DM complicated by ulcerative colitis by restoring Th17/Treg homeostasis, reducing inflammatory cell infiltration and mucosal ulceration, and thus increasing body weight (95). Ursolic acid, a natural pentacyclic triterpene, exists in a variety of Chinese medicines, such as Shanzha (Hawthorn). Ursolic acid, which belongs to the three meridians of the spleen, stomach, and liver, can reduce blood sugar and has anti-inflammatory effects. It can correct a Th17/Treg imbalance, delay the progressive destruction of pancreatic β cells, and reduce fasting blood glucose level in T1DM model rats (96, 97). Pseudostellaria heterophylla belongs to the spleen and lung meridians. Its main component, Pseudostellaria heterophylla polysaccharides, inhibited the protein expression of RORγt and increased the protein expression of FoxP3 in T2DM model rats. In addition, the STZ-induced Th17/Treg imbalance was restored, thereby significantly improving insulin resistance in T2DM model rats (98).

Other studies have found that platycodon grandiflorum, which belongs to the lung meridians, also has a certain role. Platycodin D regulates immunity; reduces the serum levels of glucose, insulin, and IL-6, in diabetic mice; significantly inhibits Th17 cells in the liver and spleen; and increases Tregs. Furthermore, by inhibiting the phosphorylation of JAK and STAT-3 and the expression of RORγt, the expression of FoxP3 increases, suggesting that Platycodin D can protect against liver injury in diabetic mice through immune regulation (99). Dongchongxiacao (Cordyceps sinensis) is a TCM belonging to the lung meridians. Through studies using NOD mice, we found that an extract of Cordyceps sinensis could slow the development of disease. This may be due to the increased frequency of Tregs and IFN-γ-producing Th1 cells in the peripheral lymph nodes (100). Yuganzi (Oil-Gan), which belongs to the meridians of the lungs and stomach, is a commonly used Chinese medicine. An extract of *Phyllanthus emblica* L., the source of Yuganzi, accelerated the decreases in blood glucose and HbA1c levels in NOD mice and reduced the levels of interleukin (IL)-1β and IL-6 in Th17 cells. It also increased the levels of IL-4, IL-10, and transforming growth factor-β1 (TGF-β1) in Th2 cells. The distribution of CD4+IL-17 and CD4+IFN-γ in CD4+ subgroup T cells was also decreased, and the distribution of CD4+IL-4 and CD4+FoxP3 was increased. Reducing the expression of these inflammatory factors in the pancreas has a protective effect on pancreatic β cells and ultimately the expression of cytokines to inhibit the development of autoimmune diabetes (101). Qingdai belongs to the liver meridian. The indirubin contained in qingdai has been confirmed to play a key role in the autoimmune response of CD4+CD25+ Tregs, which can selectively improve the levels of these cells and make the host environment more conducive to inducing immune tolerance. Qingdai has good research potential in autoimmune diseases, such as T1DM (102). Fuling (Poria), which belongs to the four meridians of the heart, lung, spleen, and kidney, is a commonly used Chinese medicine. A Poria cocos extract was shown to regulate the Th17/Treg balance, induce the release of INS, reduce blood sugar levels, and alleviate pancreatic tissue damage in T1DM model mice (103). Leigongteng (Tripterygium wilfordii) belongs to the heart and liver meridians. Its main components, tripterygium glycosides, can effectively regulate the Th17/Treg balance and may be effective in the treatment of DN. It was shown to regulate autoimmunity and inflammation by restoring the Th17/Treg balance and correcting immune imbalance (104). Qinghao (Artemisinin) inhibited the proliferation of Th17 cells in mice by increasing the number of Tregs, prolonged the survival time of islet grafts in allogeneic mice, and inhibited rejection (105). Baishao (White Peony Root), which belongs to the two meridians of the liver and spleen, contains a large amount of paeoniflorin, which reduced the levels of blood glucose, ALT, and AST in T1DM mice and inhibited lymphocyte proliferation *in vitro*. Simultaneously, it significantly increased the levels of IL-10 and CD4+FoxP3+ Tregs in T1DM mice and reduced the levels of IL-17; these activities had a remission-inducing and treatment effect on the development of T1DM (106). Ganzheye (Sugarcane Leaf), which belongs to the meridians and contains sugar leaf polysaccharides, greatly reduced the blood sugar levels of T1DM mice and improved the Th17/Treg imbalance. The medicine also improved the structure of mouse islets, reduced the apoptosis of islet β cells, increased insulin secretion, and effectively regulated immune imbalances (107). An extract of the alga Haizao (Laminaria), which belongs to the three meridians of the liver, stomach, and kidney, enhanced the levels of Tregs in T1DM rats and thus may be a new research direction for the development of DM treatments (108). In summary, different Chinese medicines belonging to various meridians can affect Treg expression, mainly by upregulating Tregs, increasing anti-inflammation, and promoting Th17/Treg balance in **Table 1**.

**Table 1:** Treg and active ingredients in Chinese medicinals.

Chinese medicinals	Attribution channel	Active ingredients	Effect on Treg
Tusizi (Dodder)	Liver, Spleen, Renal	Hyperoside	↑Promote Tregs differentiation (65)
Danshen (Salvia)	Heart, Liver	Salvia	↓Inhibition of Tregs deficient-induced autoimmunity (94)
Jianghuang (Turmeric)	Liver, Spleen	Curcumin	↑Correct Th17/Treg cells differentiation imbalance (95)
Shanzha (Hawthorn)	Spleen, Stomach, Liver	Ursolic acid	↑Correct Th17/Treg cells differentiation imbalance (96, 97)
Taizishen (Pseudostellaria Heterophylla)	Spleen, Lung	Pseudostellaria heterophylla polysaccharides	↑Correct Th17/Treg cells differentiation imbalance (98)
Jiegeng (Platycodon Grandiflorum)	Lung	Platycodin D	↑Promote Tregs differentiation (99)
Dongchongxiacao (Cordyceps Sinensis)	Lung, Renal	Cordyceps sinensis extract	↑Promote Tregs differentiation (100)
Yuganzi (Oil-Gan)	Lung, Stomach	Phyllanthus emblica L	↑Correct Th17/Treg cells differentiation imbalance (101)
Qingdai (Natural Indigo)	Liver	Indirubin	↑Increased Treg cells levels (102)
Fuling (Poria)	Heart, Lung, Spleen, Renal	Poria cocos extract	↑Correct Th17/Treg cells differentiation imbalance (103)
Leigongteng (Tripterygium Wilfordii)	Heart, Liver	Tripterygium glycosides	↑Correct Th17/Treg cells differentiation imbalance (104)
Qinghao (Artemisia Annua)	Liver, Gallbladder	Artemisinin	↑Increase Tregs, ↓decrease Th17 (105)
Baishao (White Peony Root)	Liver, Spleen	Paeoniflorin	↑Increase Tregs, ↓decrease Th17 (106)
Ganzheye (Sugarcane Leaf)	Lung, Stomach	Sugarcane Leaf Polysaccharide	↑Correct Th17/Treg cells differentiation imbalance (107)
Haizao (Algae)	Liver, Stomach, Renal	Laminaria	↑Promote Tregs differentiation (108)

“↑” means to promote, “↓” means to inhibit.

### 4.2 Tregs and Chinese medicinal compounds

Some Chinese medicinal compounds can produce different effects by modulating Tregs. The Chinese medicine Ginseng and Astragalus granules (GAG) is mainly composed of Renshen (Ginseng), Wuweizi (Schisandra), Huangqi (Astragalus), Shanyao (Chinese Yam), Dihuang (Rehmannia), Maidong (Ophiopogon), Fuling (Poria), Tianhuafen (Trichosanthin), Zexie (Alisma), Gouqi (Wolfberry), and 10 other Chinese medicines. Administration of GAG increased the number of CD4+FoxP3+ and CD8+CD122+PD1+ Tregs in the spleen and lymph nodes of NOD mice, significantly enhanced the function of islet cells, and improved glucose tolerance and insulin levels (109). The Sanhuang Xiaoyan (SHXY) recipe contains 11 Chinese herbs, including Daihuang (rhubarb), Huangqin (Scutellaria Baicalensis), Huangbo (Phelloderma Chinensis), Lianqiao (Forsythia), Gancao (Licorice), Huanglian (Coptis Chinensis), Shanzha (Hawthorn), and Huzhang (Knotweed). It reduced inflammation and edema, increased collagen synthesis, decreased the expression levels of RORγt and IL-17A, and inhibited the differentiation of Th17 cells. However, it did not affect Tregs. Regulation of CD4+ T cells was shown to significantly promote the healing of diabetic ulcers (110). Maidong (Ophiopogon), Dihuang (Rehmannia), Baishao (White Peony Root), and Maidong Dishao Decoction (MDDST), which is composed of five Chinese herbs, such as Taoren (Peach Seed) and Ziwan (Tatarian Aster Root), had a therapeutic effect on Primary Sjogren’s syndrome in NOD mice. This therapeutic effect may be a result of improving the pathological changes, alleviating the inflammatory response, significantly reducing serum levels of IL-6 and IL-17, increasing the levels of IL-10 and TGF-β, reducing the levels of Sjogren’s syndrome antigen A (SSA) and immunoglobulin G (IgG), and restoring the Th17/Treg balance (111). Keluoxin (KLX) capsules are composed of six traditional Chinese medicines: Huangqi (Astragalus), Nvzhenzi (Ligustrum), Shuizhi (Leech), Daihuang (Rhubarb), Taizishen (Pseudostellaria heterophylla), and Gouqi (Chinese Medlar). Xiao found that KLX reduced glucose reabsorption by the kidney, increased blood flow in the kidney, reduced the adverse effects of hyperglycemia on the kidney, and delayed the progression of DKD.

The mechanism of action is closely related to the upregulation of Tregs in peripheral blood (112). The YSJPTL prescription is composed of 11 Chinese herbs, including Huangqi (Astragalus), Dangshen (Codonopsis), Baizhu (White rhizome), Shanyao (Chinese Yam), Gancao (Licorice), Fuling (Poria), Heshouwu (Polygonum Multiflorum), Danshen (Salvia), and Shanzhuyu (Cornus Officinalis). YSJPTL inhibited the Notch signaling pathway, IL-17, and RORγt. Notch ligand regulates the differentiation and proliferation of Th17 cells, maintains FoxP3 expression, promotes the differentiation and proliferation of Tregs, and promotes proper Th17/Treg balance, which are involved in the improvement of renal injury in DKD mice (113). Yibu (YB) consists of 16 traditional Chinese medicines, including Huangqi (Astragalus), Dangshen (Codonopsis), Baizhu (White Rhizome), Chaihu (Bupleurum), Danggui (Dangica Sinensis), Chenpi (Tangerine Peel), Dihuang (Cooked Rehmannia), Chuanxiong (Szechwan Lovage Rhizome), Baishao (White Peony Root), and Fuling (Poria). Shen et al. showed that YB reduced FBG levels and significantly improved Th1, Th2, Th17, and Treg levels in patients with T2DM, indicating improved immune function (114). Wumei (WM) pills, which are composed of Wumei (Dark Plum Fruit), Huanglian (Coptis Coptidis), Fuzi (Aconite), Huajiao (Sichuan Pepper), Xixin (Asarum), Huangbo (Phellobium), Ganjiang (Dried Ginger), Danggui (Dangica Sinensis), and Guizhi (Cassia Twig), regulated serum levels of SFRP5, FGF21, and PTP1B, and corrected the Th17/Treg imbalance in obese patients with T2DM and poor blood glucose control, and thus improved their overall condition (115). Qikui (QK) granules, which are composed of three traditional Chinese medicines, Huangqi (Astragalus), Huangshukuihua (Yellow Hollyhoea Sunflower), and Heshouwu (Polygonum Multiflorum), inhibited the maturation of DCs, upregulated FoxP3 expression, negatively regulated the generation and differentiation of Th17 cells, promoted the generation of Tregs, regulated the Th17/Treg balance, promoted the release of the anti-inflammatory cytokine IL-10, and thus induced immune tolerance (116). Jiao et al. found that a Yuye (YY) decoction composed of Shanyao (Chinese Yam), Huangqi (Astragalus), Zhimu (Anemarrhena), Baibu (Radix Japonicae), Gegen (Pueraria Root), Wuweizi (Schisandra), and Tianhuafen (Trichosanthatis) significantly reduced the levels of Th1 and Th17 cells, and significantly increased the levels of Th2 cells and Tregs, promoted secretion from β islet cells, reduced blood sugar levels, and inhibited inflammatory reactions (117). A Gegen qinlian (GGQL) decoction composed of Gegen (Pueraria Root), Gancao (Licorice), Huangqin (Scutellaria Baicalensis) and Huanglian (Coptis) altered T2DM by changing the structure of the intestinal flora, and subsequently regulated the Th17/Treg balance, the expression of inflammatory factors and the downstream JAK-STAT/NF-κB signaling pathway in the intestinal mucosa, ultimately improving the insulin resistance of T2DM model mice (118). Jianpi Huaqing (JPHQ) is composed of Huangqi (Astragalus), Dangshen (Codonopsis), Shanyao (Chinese Yam), Huangjing (Polygonati Rhizoma), Huangqin (Scutellaria Baicalensis), Huanglian (Coptis Coptidis), and Gegen (Pueraria Root). It reduced the insulin resistance index, increased the proportion of Tregs, and reduced the proportion of Th1, Th2, and Th17 immune cells in the small intestine. It also effectively improved the insulin resistance of T2DM model rats (119).

In summary, different Chinese medicines belonging to various meridians can affect Treg expression, mainly by upregulating Tregs, increasing anti-inflammation, and promoting Th17/Treg balance. And that TCM mainly exert their therapeutic effects on DM by promoting the differentiation of Tregs, inhibiting the differentiation of Th17 cells, and correcting the Th17/Treg imbalance in **Table 2**.

**Table 2:** Treg and Chinese medicinal compound.

Chinese medicinals	Attribution channel	Effect on Treg
Ginseng and Astragalus Granules	Spleen, Lung	↑Promote Treg differentiation (109)
Sanhuang Xiaoyan Recipe	Triple energizer	↓Inhibit Th17 differentiation and do not affect Treg (110)
Maidong Dishao Decoction	Liver, Spleen, Lung	↑Promote Treg differentiation and Th17/Treg balance (111)
Keluoxin Capsule	Renal	↑Promote Treg differentiation (112)
Yishen Jianpi Tongluo Prescription	Renal, Spleen	↑Promote Treg differentiation and Th17/Treg balance (113)
Yibu Formula	Spleen, Stomach	↑Promote Treg differentiation and Th17/Treg balance (114)
Wumei Pills	Liver, Spleen	↑Promote Treg differentiation and Th17/Treg balance (115)
Qikui Granules	Spleen, Stomach	↓Inhibit Th17 differentiation and Promote Treg differentiation (116)
Yuye Decoction	Liver, Spleen, Stomach	↓Inhibit Th17 differentiation and Promote Treg differentiation (117)
Gegen Qinlian decoction	Lung, Spleen, Renal	↑Promote Treg differentiation and Th17/Treg balance (118)
Jianpi Huaqing Recipe	Spleen, Stomach, Lung	↓Inhibit Th17 differentiation and Promote Treg differentiation (119)

“↑” means to promote, “↓” means to inhibit.

## 5 Tregs and modern medical therapies

Tregs have attracted increased attention in recent years as an immunology research hotspot. In particular, more studies have been conducted to examine the Th17/Treg balance. Tregs continue to gain recognition in metabolic reprogramming. For example, Tregs, which are influenced by IL-7 and IL-15, can increase the availability of glucose and oxygen and promote glycolytic metabolism. Further improvement in Treg performance may provide better theoretical support for metabolic reprogramming by IL-7 and IL-15 for adoptive therapy (120). Sphingosine-1-phosphate (S1P) increased the number of Tregs and restored LC3 expression in Tregs. It also increased the expression levels of the mRNAs encoding Foxp-3 and S1PR1 in the submandibular glands (SMGs), ultimately improving the Sjogren’s syndrome-like symptoms in NOD model mice (121). Interestingly, N1-Methylnicotinamide (MNAM) exerts therapeutic effects by downregulating the Th17 markers RORγt and IL17A, and upregulating the Treg markers IL-10 and FoxP3. Regulating the Th17/Treg balance and reducing inflammation can reduce islet cell mortality, improve islet cell morphology, significantly reduce hyperglycemia and enhance insulin secretion, providing a theoretical basis for new strategies to treat T2DM. These results also show that Tregs are plastic and dynamic (122, 123). Studies have confirmed that IInsB-g7 CAR can redirect Treg-specific function in NOD mice, enhance the inhibitory effect of insulin b10–23 peptide stimulation, and prevent BDC2.5 T cell adoptive metastasis in diabetes. Therefore, Treg adoptive immunotherapy is a promising approach to prevent or treat T1DM (124). Nanotechnology can also be used to promote immune tolerance through systemic, *in vivo* expansion of antigen- and disease-specific Tregs or by inhibiting the loss of systemic or local antigen-presenting cells and antigen-specific T cells (125). Intravenous infusion of C-C motif chemokine receptor 2-engineered mesenchymal stromal cells was shown to reshape the inflammatory properties of macrophages, inhibit monocyte infiltration, and promote Treg accumulation at an injury site (126). Estradiol enhanced the differentiation function of FoxP3+ Tregs in NOD mice and improved the protective effect of α-galactoceramide on female NOD mice, suggesting that the estrogen/invariant natural killer T cell axis as a potential new target against the onset of diabetes at the stage of insulitis (127). Fenofibrate, a synthetic ligand of peroxisome proliferator-activated receptor α (PPAR-α), can activate the PPAR-α/LXR-β signaling pathway. The biological effect of regulating the Th1/Th17/Treg cell response in NOD mice through treatment with fenofibrate was a reduction in the relevant inflammatory indicators (128). During Treg differentiation, the number of mitochondria and the levels of FoxP3 expression both increase. TGF-β1 can also promote PGC-1α-mediated mitochondrial fusion, promote metabolic reprogramming from glycolysis to fatty acid oxidation by inhibiting the expression of HIF-1α, and promote the formation of Tregs (129, 130). In the process of autoimmunity, functional changes in Tregs are closely related to dysfunction of mitochondrial autophagy, enhancement of the DNA damage response, and mitochondrial oxidative stress (131). Studies have shown that valproic acid increases the expression of FoxP3, a key transcription factor that controls the development and function of Tregs, promotes the differentiation of Tregs, and effectively prolongs the survival time of islet grafts. This study provides evidence for the potential mechanism underlying *in vitro* adoptive transfer of VPA-induced Treg cell therapy to inhibit autoimmune recurrence and using Valproic acid to treat isogenic islet transplantation in T1DM (132). Alpha-lipoic acid prolonged the survival of islet grafts in NOD mice and reduced the incidence of diabetes, which may be related to a decrease in number of Th1 cells and an increase in the number of Tregs. In addition, Alpha-lipoic acid also significantly improved the differentiation of Tregs *in vitro*. Therefore, it has potential for use in islet transplantation in T1DM and Treg-based therapy (133). PDK1 also regulated the survival of Tregs by controlling REDOX homeostasis and effectively increasing peripheral tolerance (134). Overall, the plasticity and dynamic regulatory functions of Tregs make them an important research focus, especially for immune tolerance and autoimmune diseases.

## 6 Conclusion

Tregs, which have unique transcriptomes, growth patterns, survival factors, and T cell receptor (TCR) repertoires, are an important cell population in the adaptive immune response. They play key immunomodulatory roles and function to maintain immune homeostasis, restore immune tolerance, and inhibit immune overreactions and inflammatory responses (135, 136). Tregs are attractive candidates for treating inflammatory diseases, autoimmune diseases, and transplant rejection (137, 138). The immune disorder in DM is closely related to Treg dysfunction, and restoring the function of Tregs is considered an effective therapeutic strategy for improving immunological diseases, such as DM and its complications (139). TGF-β is the biological core of immunosuppressive Tregs and pro-inflammatory, pathogenic, and immunomodulatory Th17 cells (140). Tregs play a crucial role in maintaining the immune tolerance of β islet cells and can reduce autoimmune reactions by producing anti-inflammatory cytokines such as IL-10 and TGF-β to inhibit the overactivation of effector T cells (25). For example, promoting the expansion or functional recovery of Tregs through specific immune interventions can help re-establish immune tolerance, delay the progression of T1DM, and reduce autoimmune damage to β cells. It can also regulate the expression of FoxP3, which is a marker of Tregs. Because FoxP3 is in a key position in the transcription factor network and the Treg-specific epigenetic landscape, it may be possible to use antigen-specific conventional T cells to prepare functionally stable FoxP3-expressing Tregs to treat autoimmune diseases and further understand the development and function of Tregs (141, 142). The inflammatory environment restricts the immunosuppressive activity of Tregs and alters the differentiation of both Tregs and related tissue cell subsets (143). Oncostatin-M (OSM) blocks the differentiation of adipocyte precursors. When the genes for OSM (especially in Tregs) or OSM receptors (especially in stromal cells) are deleted, insulin sensitivity and related metabolic markers are severely impaired (144). Tregs not only improve insulin sensitivity by inhibiting the inflammatory response, but also slow down the progression of T2DM by regulating metabolic dysfunction (55).

Tregs exert a protective effect against kidney disease by inhibiting local immune responses and reducing inflammation and fibrosis in the kidney. In addition, regulation of the Th17/Treg balance can inhibit the infiltration of immune cells into the kidney, control proteinuria, and reduce kidney injury60. Therefore, functional restoration of Tregs may be a therapeutic strategy for DN. Tregs can slow down damage to the retinal microvessels through their anti-inflammatory effects, effectively inhibiting the inflammatory response of retinal endothelial cells, reducing pathological angiogenesis, and delaying the progression of lesions. Patients with diabetes often have vascular lesions such as atherosclerosis. Diet-induced dyslipidemia also promotes the migration of Tregs to the inflammatory peritoneum and atherosclerotic lesions *in vitro* (145). However, Tregs have a significant protective effect and can promote vascular repair by inhibiting the inflammatory response of the vascular endothelium. This is achieved by regulating the proportion of M2-type macrophages to slow down inflammation and structural damage to the blood vessels, thereby slowing the progression of atherosclerosis and reducing the incidence of cardiovascular complications. Tregs also improve nerve function by regulating immune responses, reducing nerve damage, and relieving oxidative stress.

The multi-target, low-toxicity characteristics of TCM make them an important adjuvant therapy for the treatment of DM and its complications. TCM have a unique advantage for immunotherapy in DM, especially in regulating the function of Tregs (146). Many ingredients in TCM improve the immune imbalance in DM by regulating the immune system and restoring Treg function. For example, ingredients such as Cuscuta, Salvia miltiorrhiza, and turmeric have been shown to inhibit inflammation and alleviate immune damage in DM by increasing both the number of Tregs and their immunosuppressive function. With the progress in modern immunology and molecular biology, the plasticity and dynamic regulatory functions of Tregs have been widely studied and have become important targets for DM immunotherapy (147). Studies have suggested that single-nucleotide polymorphisms (SNPs) associated with common autoimmune diseases are mainly concentrated in CpG-demethylated regions that exist specifically in naïve Tregs. Therefore, naïve Treg-specific CpG hypomethylation plays a key role in controlling Treg-specific gene transcription and epigenetic modification (148). Strategies for restoring Treg cell function through metabolic reprogramming, immune cell therapy, and genetic engineering have been actively explored. For example, IL-7 and IL-15 enhance the immunosuppressive function of Tregs by regulating their metabolism and have been shown to effectively increase the number and function of Tregs, thereby improving the immune imbalance in DM. In addition, drugs such as valproic acid and alpha-lipoic acid reduce the immune inflammatory response in DM by promoting the functional recovery of Tregs (132, 133). Bcl10, which acts as a scaffolding protein in the Carma1-Bcl10-Malt1 (CBM) complex, plays a key role in signal transduction. Bcl10 further increased the expression of the transcription factors T-bet and HIF-1α, and Tregs were transformed into pro-inflammatory cells that produce IFNγ, suggesting that Bcl10 is also necessary for the development and function of Treg cells (149). The cell-state spectrum of Tregs stimulated by IL-6 was studied using single-cell RNA sequencing, and IL-6–stimulated Tregs were divided into two subgroups. However, Tregs deficient in cytochrome P450 family 1 subfamily A member 1 (CYP1A1) showed a Th17-like phenotype after IL-6 stimulation, suggesting CYP1A1 as a potential Treg regulator with biotherapeutic clinical applications (150). Ubiquitin-specific peptidase 1 (USP1) can also inhibit the differentiation of Th17 cells and promote the differentiation of Tregs, which plays a key role in regulating adaptive immune responses (151). Future studies should not only reveal the detailed mechanism of action of Tregs in DM and its complications but could also lead to the development of new immune interventions, using techniques such as gene editing and nanotechnology, to regulate the differentiation of Tregs and enhance their function with an aim to improve the immune tolerance and self-repair ability of patients with DM (152). Using a multidimensional approach to explore Treg biology will lead to a more refined understanding of Treg cells biology and new therapeutic approaches based on tissue-specific functions (153, 154).

In summary, the immunoregulatory role of Tregs in DM and its related complications is at the core of diabetes immunotherapy (155). Regulating the function of Tregs would not only restore immune homeostasis and reduce the inflammatory response but also effectively alleviate the progression of DM and its complications. However, the existing studies still have some limitations: Firstly, Tregs are prone to phenotypic instability or functional loss in high inflammation or metabolic stress microenvironments, resulting in a decline in inhibitory effect; Secondly, peripheral-expanded or *in vitro*-induced Tregs may have difficulty maintaining their functions in the body for a long time, and lack precise regulatory means for specific tissues; Thirdly, significant differences in immune background and metabolic status among different individuals lead to large individual fluctuations in immune intervention effects; Finally, current clinical translation lacks efficient and safe strategies to precisely regulate the function of Tregs. Therefore, future research should focus more on the exploration of the stability of Treg function, metabolic adaptability, and tissue-specific regulatory mechanisms, while combining strategies such as biomaterial delivery systems, small molecules, or traditional Chinese medicine intervention to achieve precise and controllable immune intervention. The author believes that simply pursuing an increase in the number of Tregs may not achieve long-term efficacy. The future treatment direction should shift from “increment” to “quality control”, by optimizing the function, stability, and plasticity of Tregs, to achieve efficient, safe, and individualized immunotherapy for diabetes and its complications. This not only provides new theoretical basis for diabetes immunotherapy, but also lays the foundation for the development of more precise intervention strategies in the future.
